# Electromyography patterns during running in injured versus healthy runners: a systematic review

**DOI:** 10.1186/s12891-026-10064-z

**Published:** 2026-07-09

**Authors:** Joseph Liddy, Stephen Preece, Bradley Stephen Neal, Christopher Bramah

**Affiliations:** 1 School of Health & Society, University of Salford, Salford, UK; 2 Sports and Exercise Medicine, Queen Mary University of London, London, UK

**Keywords:** Electromyography (EMG), Running, Running related injuries, Biomechanics, Muscle activation

## Abstract

**Background:**

Running injuries are frequently linked to biomechanical factors. Although there has been substantial research investigating the link between kinematic factors and running injury, there has been comparatively less focus on the role of muscle coordination. Previous literature has identified that altered EMG patterns may contribute to the overloading of tissues and to the development or persistence of injury. Therefore, the aim of this systematic review was to synthesize previous research comparing Electromyography (EMG) patterns between injured (Patellofemoral Pain, Achilles Tendinopathy, Iliotibial Band Syndrome, Medial Tibial Stress Syndrome and Hamstring Strain Injury) and uninjured runners.

**Methods:**

Six databases (CINAHL, MEDLINE, ProQuest, Scopus, Google Scholar, and Web of Science) were searched from inception to February 2025. Included studies assessed muscle activation via EMG during treadmill or overground running or sprinting. Methodological quality was rated independently by two reviewers using a modified Downs and Black checklist. Meta-analyses were deemed appropriate if more than two studies measured the same outcome parameter. Seventeen studies were included.

**Results:**

Strong evidence indicated no consistent differences in Gluteus Maximus or Gluteus Medius amplitude, or in the onset and duration of Gluteus Medius, Vastus Medialis Oblique, or Vastus Lateralis activity between runners with patellofemoral pain and uninjured runners. These conclusions were based on pooled analyses of relevant subsets of patellofemoral pain studies for each outcome, rather than the full body of included literature. Furthermore, strong evidence indicated no differences in Gluteus Medius amplitude, onset and duration in all running injury populations compared to controls. These conclusions were based on pooled analyses of relevant subsets studies that measured Gluteus Medius for each outcome.

**Conclusions:**

These findings suggest neuromuscular deficits are not present across or within specific injury types (Patellofemoral Pain, Achilles Tendinopathy, Iliotibial Band Syndrome, Medial Tibial Stress Syndrome and Hamstring Strain Injury). The findings may reflect methodological inconsistencies across studies, such as electrode placement, EMG normalisation, task design, and participant characteristics. Future research should adopt standardised EMG protocols, consider the role of the entire kinetic chain, and thoroughly report participant running profiles.

**Supplementary Information:**

The online version contains supplementary material available at 10.1186/s12891-026-10064-z.

## Main findings

1. Gluteus Medius was the most frequently studied muscle across running related injuries. Meta-analysis indicates strong evidence of no difference in amplitude or timing of muscle onset between injured and uninjured runners.
2. Patellofemoral pain was the most frequently studied injury. Meta-analysis indicated strong evidence of no difference in the amplitude of Gluteus Maximus or Gluteus Medius activity, and no difference in the onset or duration of Gluteus Medius, Vastus Medialis Oblique or Vastus Lateralis activity.
3. Substantial methodological heterogeneity exists across studies. Including variations in running protocols, EMG normalisation and electrode placement, which likely contributes to inconsistent findings and highlights the need for more standardisation in data collection protocols, analysis techniques and reporting.
4. Limited studies report combined EMG with kinetic and kinematic data which further limits our understanding of the how muscle activation patterns respond to external forces.

## Introduction

Running is one of the most popular forms of physical activity, but one that is associated with high injury rates [1]. A recent systematic review reported an overall injury incidence of 40.2% and a prevalence of 44.6%, highest in the knee, lower leg and ankle [2]. Patellofemoral Pain (PFP), Iliotibial Band Syndrome (ITBS), Medial Tibial Stress Syndrome (MTSS), and Achilles Tendinopathy (AT) are among the most frequently reported injuries in the literature, with prevalence rates ranging from 7% to 16% [3–6]. The high prevalence of these conditions highlights the persistent burden they place on runners and reinforces the importance of developing effective injury prevention and rehabilitation strategies to support long-term running participation.

Running injuries are multifactorial, but altered biomechanics represent one factor frequently associated with injury [7], by influencing tissue stress and strain during running [8]. Although there is a large body of work on kinematic and kinetic factors [9–14], muscle activation patterns are comparatively less understood. Injury-induced pain has been reported to alter muscle activation patterns, but with both decreased and increased motor unit discharge rates demonstrated [15]. This heterogeneous muscle response to pain highlights a complex muscle-specific relationship with neuromuscular control.

Altered muscle activation has been implicated in the development and persistence of injury. For example, lower activity in both the oblique and gluteus maximus muscles is prospectively associated with the development of hamstring injuries in footballers [16], whilst decreased biceps femoris (BF) muscle activity has been observed following hamstring injury [17, 18]. Altered neuromuscular patterns have also been reported after anterior cruciate ligament reconstruction, with increased co-contraction of the quadriceps and hamstrings observed [19, 20]. Runners with AT have reported increased tibialis anterior (TA) activity during push-off and decreased lateral gastrocnemius (LG) activity during the weight acceptance phase [21, 22]. These adaptations, though potentially protective in the short term, may lead to long-term dysfunction or re-injury and contribute to the development and/or persistence of running-related injuries [23].

Existing systematic reviews investigating electromyography (EMG) in running-related injuries have focused on specific conditions rather than a broad spectrum of injuries. A systematic review evaluating gluteus medius (GMed) function reported shorter duration of GMed activity during stance in runners with PFP, with limited evidence suggesting similar alterations in runners with AT [24]. However, while it is possible that adaption to pain may lead to localised muscle changes, it is also possible that there could be deficits in muscle coordination which play a role in the development or persistence of injury. Importantly, several running injuries have been shown to exhibit overlapping kinematic and kinetic characteristics, including altered joint moments and changes in proximal segment control during running [9–13]. These observations may suggest the presence of common alterations in muscle coordination which may either contribute to the kinematic/kinetic factors or, alternatively, lead to tissue overload at multiple anatomical locations and therefore predispose to different forms of running injury.

No previous systematic review has synthesised EMG characteristics from multiple lower limb muscles across range of running injury types. Therefore, this review aimed to synthesise available EMG data of lower limb muscles from injured and uninjured runners to gain insight into potential shared mechanisms for the development or persistence of running injury. The specific objectives were to compare EMG amplitude, timing, and duration of muscle activity between injured and uninjured runners, both across multiple injury types and within specific injuries, to pool evidence using meta-analysis where possible, and to evaluate the methodological quality of included studies.

## Methods

This review was registered a priori with PROSERO (CRD42024622979) and conducted in accordance with the preferred reporting items for Systematic Reviews and Meta-Analysis (PRISMA) statement [25]. The sole deviation from the registered protocol was the addition of an author following registration.

### Search strategy

Electronic database searches were conducted in MEDLINE (via PubMed), CINAHL, Scopus, Web of Science, ProQuest, and Google Scholar from database inception to February 2025. Database-specific search strategies were developed using a combination of controlled vocabulary (e.g. MeSH and CINAHL Headings) and free-text keywords related to electromyography and running. Searches were restricted to title and abstract fields where applicable. No restrictions were placed on running speed. Searches were limited to human adult populations and English-language publications. The complete search strategies for each database, including field restrictions and applied filters, are provided in Supplementary File 1.

### Selection criteria

Eligible case-control and prospective studies were required to assess muscle activation via EMG during treadmill or overground running or sprinting, and were included regardless of speed specifications. Running and sprinting studies were both included due to the limited volume of EMG research in injured running populations, as restricting inclusion to submaximal running would have substantially reduced the available evidence base. Studies were excluded if they solely examined EMG patterns during walking, cutting manoeuvres, obstacle navigation, or stair ambulation. Populations targeted were male and/or female adults with running-related injuries (Patellofemoral Pain, Achilles Tendinopathy, Iliotibial Band Syndrome, Medial Tibial Stress Syndrome and Hamstring Strain Injury) and healthy groups. All identified records were collated in Endnote (v20.4.1, Clarivate Analytics; Philadelphia, PA, USA) and uploaded to Covidence review management software (Veritas Health Innovation; Melbourne, Australia). Covidence automatically removed identified duplicates, with any remaining removed manually during screening. Titles and abstracts were first screened by two independent reviewers (JL and CB) against the inclusion and exclusion criteria, followed by remaining full text articles. Reference list searching and citation tracking using Google Scholar was also completed for all included studies. All disagreements were handled by a third reviewer (SP). All included articles were published in English.

### Data extraction

One author (JL) independently extracted relevant data from the included studies before being checked for accuracy by a second reviewer (CB). Extracted data included the injury condition and comparison of participant groups, participant demographics (age, body mass, height, weekly running distance, years of running experience), running protocol (e.g., treadmill or overground, running speed) and EMG protocol, including muscles where EMG was applied (bilaterally or unilaterally), whether EMG was surface or fine wire, electrode placement, normalisation methods, signal processing in terms of filter frequency, sampling frequency, and window/technique. Both muscle temporal activity (muscle activity onset/offset and duration) and amplitude were extracted if reported to enable for a complete report of all EMG parameters.

### Quality assessment

A modified version of the Downs & Black Quality Index was used to evaluate the quality of included studies [26], considering only questions relevant to non-randomised studies [27, 28]. This version includes a total of 15 questions with a maximum overall score of 16 [27]. Studies scoring *≥* 11 were considered high quality (HQ), between 6 and 10 moderate quality (MQ), and of *≤* 5 low quality (LQ). Two independent raters (JL & CB) evaluated each study with discrepancies resolved at a consensus meeting.

### Data analysis

Means and standard deviations for individual muscles and EMG parameters assessed (e.g., amplitude or time of onset etc.) were used to calculate standardised mean differences (SMD) and standard error (SE). Where data could not be extracted from the published manuscript, authors were contacted a maximum of twice to request access to relevant data. If relevant data were only presented in figures, WebPlotDigitizer (v5, Automeris LLC) was used to extract relevant data points.

Random-effects meta-analyses were conducted using JASP (version 0.19.3.0). Data were pooled when > 2 studies reported the same muscle and EMG parameter (amplitude, onset, offset, duration, or integral) with sufficient methodological homogeneity (e.g., comparable normalisation procedures and phase definitions). For analyses exploring patterns across injury types, pooling was only undertaken when data were available from ≥ 2 distinct injury groups. If fewer than two injury types were represented, meta-analysis across injury types was not performed. The level of statistical heterogeneity for pooled data was established using *I²* statistics (heterogeneity defined as I² > 50%, *p* < .05) [29]. Pooled SMDs were interpreted as 0 to 0.19 = trivial, 0.2–0.49 = small, 0.5 to 0.79 = medium and ≥ 0.8 = large [30].

### Strength of evidence

Overall strength of evidence was interpreted based on the van Tulder criteria [31], applied as follows:

- Strong evidence: Consistent findings in ≥ two HQ studies.
- Moderate evidence: Findings from one HQ study or consistent findings across multiple MQ or LQ studies.
- Limited evidence: Findings from one MQ or LQ study.
- Conflicting evidence: Inconsistent results across multiple studies regardless of quality.

## Results

### Study selection

**Fig. 1:**
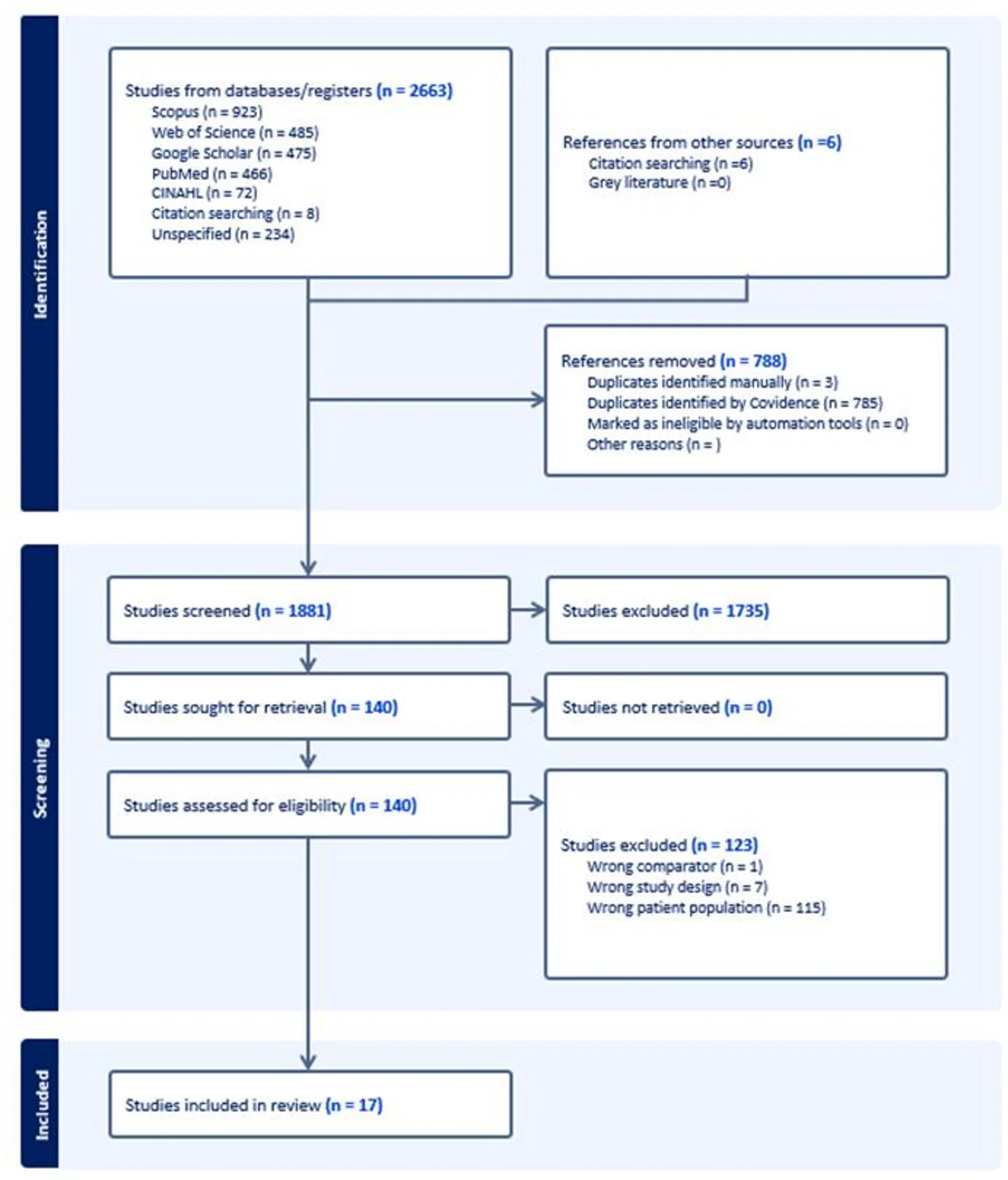
PRISMA flow diagram produced through Covidence. Figure displays details of initial search, screening and quantity of studies included for the review

The initial search identified 2,663 articles, and 17 studies met the eligibility criteria following screening of titles and abstracts, full texts, and citation searching. All eligible studies were published in English (Fig. 1).

### Study characteristics

Study and participant characteristics are described in Table 1. Six studies included participants with PFP [32–37], six included participants with AT [38–43], and three included participants with ITBS [44–46]. Single studies included participants with MTSS [47] and Hamstring Strain Injuries (HSI) [48]. Of the seventeen studies included, sixteen used a retrospective design [32–46, 48] and one used a prospective design [47]. Eleven studies reported the weekly running volume of the participants [33–36, 39, 41–46], three reported the years of running experience [33, 35, 39], and eight reported runner type/performance level (e.g., novice, recreational) [33–35, 38, 40, 44, 47, 48]. Sixteen studies included an injured group and a control group [32–47] and one study compared the injured leg to the uninjured leg [48].

**Table 1:** Summary of study designs, sample characteristics, injury type and participant demographics of included studies

Author/Year	Study Design	Condition / Comparison	Sample Size	Demographics Uninjured	Demographics Injured (+ diagnostic criteria)	Runner Type (novice/rec/elite/sprinter)	Experience (yrs)	Weekly Vol. (km)	Footwear	Foot Strike Pattern
Azevedo et al., 2009 [40]	Case-control	Achilles Tendinopathy	42 (32 M, 10 F) Uninjured: 21	Age: 38.9 ± 10.1· Ht: 1.74 ± 8· Mass: 70.2 ± 10.9	Age: 41.8 ± 9.7· Ht: 1.78 ± 7.4· Mass: 77.6 ± 12.6· Pain/stiffness/tenderness over tendon.	Not reported	≥ 3 yrs	> 15	Standardized shoes	RFS
Baker et al., 2018 [44]	Cross-sectional	Iliotibial Band Syndrome	30 (16 M, 14 F) Uninjured: 14 Injured:15	M Age: 31.1 ± 6.0· F Age: 31.4 ± 7.5· Mass: 77.1 ± 11.4/65.9 ± 7.8	M Age: 32.7 ± 5.9· F Age: 33.4 ± 7.5· Mass: 78.2 ± 7.4/62.8 ± 8.9· Noble compression test positive.	Mixed runners	Not reported	> 16.1	Participant’s own	Not reported
Baur et al., 2004 [41]	Case-control	Achilles Tendinopathy	22 (sex not reported) Uninjured: 14 Injured: 8	Age: 36 ± 9· Ht: 1.79 ± 5· Mass: 73 ± 8	Combined values with G1 for demographics. Diagnostic: Pain/tenderness over Achilles tendon.	Recreational	Not reported	Not reported	Barefoot-like shoe & reference shoe	RFS
Baur et al., 2011 [42]	Case-control	Achilles Tendinopathy	60 (40 M, 20 F) Uninjured: 30 Injured: 30	Age: 37 ± 10· Ht: 1.74 ± 0.1· Mass: 67 ± 13	Age: 41 ± 7· Pain localised to 2–6 cm from insertion· Training > 32 km/wk.	Not reported	Not reported	G1: 42 ± 14	Neutral shoes	RFS
Besier et al., 2009 [32]	Cross-sectional	Patellofemoral Pain	43 (19 M, 24 F) Uninjured: 16 Injured: 27	M Age: 27.2 ± 3· F Age: 28.8 ± 4.7· Mass: 74.2 ± 4.2/58.3 ± 4.6	M Age: 30.5 ± 4.5· F Age: 28.7 ± 4.6· Mass: 72.4 ± 12.5/62.7 ± 10· Pain during daily activities.	Not reported	Not reported	Not reported	Not reported	Not reported
Brown et al., 2019 [45]	Case-control	Iliotibial Band Syndrome	32 Female Uninjured: 20 Injured: 12	Age: 28.9 ± 6.1· Ht: 1.6 ± 0.09· Mass: 56.8 ± 5.2	Age: 32.4 ± 7.9· Ht: 1.7 ± 0.06· Mass: 60.6 ± 5· Physician/Physiotherapist confirmed.	Not reported	Not reported	> 24.1	New Balance shoes	RFS
De Almeida Britto et al., 2021 [33]	Case-control	Patellofemoral Pain (Unilateral/Bilateral)	32 Male Uninjured: 20	Age: 27 ± 1.7· Ht: 1.74 ± 0.08· Mass: 74.7 ± 8.3	Age: 28.7 ± 4.3· Ht: 1.76 ± 0.04· Mass: 72.5 ± 7.1· Pain during daily activities· No treatment during test period.	Recreational	9.93 ± 5.37	27.05 ± 12.12	Participant’s own	Not reported
Esculier et al., 2015 [34]	Cross-sectional	Patellofemoral Pain	41 (10 M, 31 F) Uninjured: 20 Injured: 21	Age: 33.2 ± 6· Ht: 1.69 ± 0.071· Mass: 62.8 ± 9.4	Age: 34.1 ± 6· Ht: 1.68 ± 0.067· Mass: 67.4 ± 11.5· Pain ≥ 3 months duration.	Recreational	Not reported	24 ± 10.9	Maximalist/Minimalist shoes	RFS (G1: 70%, G2: 67%)
Foch et al., 2020 [46]	Cross-sectional	Iliotibial Band Syndrome	30 Female Uninjured: 15 Injured:15	Age: 25.1 ± 7· Ht: 1.66 ± 0.06· Mass: 58.5 ± 6.5	Age: 26.7 ± 9.3· Ht: 1.68 ± 0.07· Mass: 61.4 ± 7.1· Pain-free for ≥ 1 month.	Not reported	Not reported	34.2 ± 23.1	Neutral shoes	Not reported
Higashihara et al., 2019 [48]	Cross-sectional	Hamstring Strain Injury (Unilateral)	10 Male Previously injured	Age: 19.9 ± 0.3· Ht: 1.72 m ± 3.9· Mass: 65.9 ± 7.1	Time post-injury: 27.3 ± 20.5 months. Diagnostic: History of unilateral strain with training exclusion for ≥ 1 week.	Collegiate Sprinter	Not reported	Not reported	Not reported	Not reported
Muniz et al., 2023 [35]	Cross-sectional	Patellofemoral Pain	31 Male Uninjured: 18 Injured: 13	Age: 28.9 ± 4.4· Ht: 1.7 ± 0.1· Mass: 74.1 ± 7.8	Age: 27.8 ± 3.1· Ht: 1.8 ± 0.1· Mass: 75.7 ± 10.2· Pain ≥ 2 months.	Recreational	G1: 9.7 ± 5.7	28.4 ± 13.3	Neutral shoes	Not reported
Naderi et al., 2020 [47]	Prospective Case-control	Medial Tibial Stress Syndrome	123 healthy (sex not reported) Uninjured: 89 Injured: 23 (not all followed up/ developed different injuries)	Age: 20–30· Ht/Mass: Not reported	Age: 20–30· Pain confirmed by sports physician within 17-week follow-up.	Phys. Ed. students	Not reported	Not reported	Orthoses	Not reported
Pingel et al., 2013 [38]	Case-control	Achilles Tendinopathy	18 (10 M, 8 F) Uninjured: 9 Injured: 9	Age: 53 ± 9· Ht: 1.80·0· Mass: 81·17	Age: 54 ± 9· Diagnostic: Chronic Achilles irritation for > 6 months.	Not reported	Not reported	Not reported	Not reported	Not reported
Smith et al., 2013 [43]	Case-control	Achilles Tendinopathy	33 Male Uninjured: 17 Injured: 14	Age: 37 ± 8· Ht: 1.79 ± 0.6· Mass: 77.4 ± 10.2	Age: 43 ± 8· Ht: 1.79 ± 0.5· Mass: 82.3 ± 11.1· Ultrasound-confirmed midportion tendinopathy.	Not reported	Not reported	G1: 37.6 ± 16.4	Nike sandals	Not reported
Souza & powers, 2009 [37]	Cross-sectional	Patellofemoral Pain	41 Female Uninjured: 20 Injured: 21	Age: 26 ± 5· Ht: 1.7 ± 6· Mass: 62.9 ± 6.6	Age: 27 ± 6· Ht: 1.7 ± 8.1· Mass: 64.7 ± 10.4· Pain ≥ 3 months duration.	Not reported	Not reported	Not reported	New Balance shoes	Not reported
Willson et al., 2011 [36]	Cross-sectional	Patellofemoral Pain	40 Female Uninjured: 20 Injured: 20	Age: 21.6 ± 4.5· Ht: 1.69 ± 0.09· Mass: 62.1 ± 8.9	Age: 21.3 ± 2.6· Ht: 1.68 ± 0.06· Mass: 62.9 ± 7.7· Pain ≥ 2 months duration.	Not reported	Not reported	5 ± 3.6	New Balance shoes	Not reported
Wyndow et al., 2013 [39]	Case-control	Achilles Tendinopathy	34 Male Uninjured: 19 Injured: 15	Age: 36 ± 8· Ht: 1.79 ± 0.05· Mass: 77 ± 10	Age: 42 ± 7· Ht: 1.77 ± 0.05· Mass: 80 ± 11· Symptomatic ≥ 3 months.	Recreational	Not reported	40 ± 16	Nike sandals	Not reported

### Methodological quality

Scores across included studies ranged from 4 to 14 out of a maximum 16 (See Supplementary File 2). Eight studies were rated as HQ [32–36, 40, 43, 47], eight were MQ [37–39, 44–46, 48] and one was LQ [41]. Inter-rater reliability was calculated using percentage agreement for all studies and the median agreement was 76.5%, with an interquartile range of 50% to 97%. Item 21 demonstrated the lowest agreement of 30%, with 4 items achieving 100% agreement.

### Running protocol

Running protocols are detailed in Table 2. Ten studies used treadmill trials [32–35, 37, 40, 41, 43, 44, 46] while seven used laboratory tracks [36, 37, 39, 42, 45, 47, 48]. Five studies used self-selected speeds [32, 38, 39, 43, 46], eleven used pre-determined speeds [33–37, 40–42, 45, 47, 48] and one transitioned from pre-determined to self-selected after five minutes [44]. Only one study involved maximal speed [48]; all others used submaximal conditions [32–47]. Trial count per participant varied: seven studies did not report this variable [32–35, 40, 43, 44] while others reported between three and ten trials [36–39, 41, 42, 45–48]. In eight studies, trial distances were pre-determined [36–39, 42, 45, 47, 48] whereas the nine treadmill-based studies did not specify a target distance [32–35, 40, 41, 43, 44, 46].

**Table 2:** Summary of electromyography measurement protocols, signal processing methods, normalisation methods and principal findings for injured versus uninjured groups

Author/Year	Running Protocol	EMG Protocol	Signal Processing	EMG Results (injured compared to controls)	Other Biomechanical Findings
Azevedo et al., 2009 [40]	10 m track (10 trials, self-selected speed) Average speed Uninj: 3.00 ± 0.41 m/s Average speed Inj: 2.97 ± 0.37	TA, LG, PL, RF, BF, GMed (surface, midpoint of belly)	Filtering: High Pass (15 Hz) Smoothing: RMS (50 ms) Normalisation: Mean across gait cycle Outcome: Integrated EMG across specific gait phases	↓ TA pre-strike activation in inj (*p*=.003). ↓ GMed and RF post-strike activation in inj (*p*=.004 & *p* = .000)	Knee ROM ↓ in injured group (*p*=.011); impact forces similar.
Baker et al., 2018 [44]	30-min treadmill run (2.74 m/s first 5 min, then self-selected) Average speed not reported	TFL, GMed, GMax (surface, anatomical landmarks)	Filtering: High pass (20 Hz) Smoothing: Rectified, low pass filtered and RMS (1 s) Normalisation: MVIC Outcome: Mean EMG during the deceleration phase of stance	TFL activation ↑ in injured at 3 min (*p* = .02). NS at 30 min. GMAX NS at 3 min, and at 30 min. From 3 to 30 min GMAX activation ↑ in injured (*P* = .09).	ITBS ↑ KADD at 30 min compared to control (*p* = .002).
Baur et al., 2004 [41]	Treadmill run completed over 20 consecutive stride cycles (3.33 m/s) Average speed not reported	TA, PL, LG, MG, SOL (surface, placement not detailed)	Filtering: Not described Smoothing: Rectified & moving average (50 ms) Normalisation: Mean across gait cycle Outcome: Muscle on/off > 10% signal max & mean across specific gait phases	The amplitude of EMG in the extensor loop during the weight acceptance was ↑ in uninj runners compared to AT patients, in both barefoot and shod conditions (*p* = .00)	No significant differences in centre of pressure between conditions.
Baur et al., 2011 [42]	5-min treadmill (3.33 m/s), 10 trials at the end Average speed not reported	TA, PL, MG (bilateral, placement not detailed)	Filtering: High pass (10 Hz) Smoothing: Rectification and averaging across 10 strides Normalisation: Mean across gait cycle Outcome: Muscle on/off > resting signal + 2 SD & mean across specific gait phases	↓ PL activation in AT group weight acceptance phase (*p*<.05). ↓ MG activation in AT runners during weight acceptance and push-off.	N/A
Besier et al., 2009 [32]	Treadmill with distance or time of run not reported (self-selected speed) Average speed uninjured: 2.67 ± 0.29 m/s males, 2.64 ± 0.36 females Average speed injured: 2.65 ± 0.23 males, 2.65 ± 0.28 females	VL, VMO, TA (surface, SENIAM)	Filtering: High pass (30 Hz) Smoothing: Rectified & low pass filter (6 Hz) Normalisation: MVIC Outcome: Co-contraction & EMG-driven model to estimate peak force	Qualitatively, the level of co-contraction during running remained higher throughout the stance phase compared to walking (up until 60% of the stance phase).	PFP subjects produced 13% ↓ pKE moment (*p*=.041)
Brown et al., 2019 [45]	30 m lab runway (5 trials, 3.35 m/s) Average speed uninj: 3.2 ± 0.29 Average speed inj: 3.1 ± 0.44 m/s	GMed, TFL (surface, anatomical landmarks)	Filtering: High pass (10 Hz) Smoothing: Rectified & low pass filter (20 Hz) Normalisation: None Outcome: Muscle on > resting mean + 10% (trial max – resting mean)	GMed/TFL NS timing.	↓ fatigue resistance in GMed observed in injured group. Injured runners had ↓ GMed strength (*p*<.01).
De Almeida Britto et al., 2021 [33]	46 m on treadmill (3.06 m/s) Average speed not reported	VMO, VL, GMed (surface, SENIAM)	Filtering: High pass (20 Hz) Smoothing: Rectified & low pass filter (7 Hz) Normalisation: Max across 15 s trial Outcome: Muscle on/off TKEO > 3 SD resting mean for > 10 ms, duration	VMO delayed onset (*p* = .023), ↓ duration (*p* = .030), ↓ duration integral (*p* = .004), preactivation level ↓ (*p* = .029) and after heel strike level ↓ ( *p*= .041). GMed: Delayed onset (*p* = .024) & duration (*p* = .024)	N/A
Esculier et al., 2015 [34]	5-min treadmill (2.22–2.78 m/s) Average speed not reported	GMed, GMax, VMO, VL, SOL (surface, SENIAM)	Filtering: High pass (30 Hz) Smoothing: Rectified Normalisation: Dynamic segment weight tasks Outcome: Muscle on/off > 5 SD resting mean for > 25 ms, duration, peak activation & mean across specific gait phase	Longer SOL activation in injured group (*P* = .031) . NS differences in GMed, GMax, VL and VMO peak activation, mean activation, onset and duration. PFPS RFS ↓ Gmed peak (*P* = .049) & mean activation (*P* = .005) v RFS controls.	NS differences in HADD or IR between groups. ↑ pHADD at toe off (*P* < .001), ↑ HADD excursion (*P* = .012) between mid-stance to toe off in PFPS. ↑ pHADD at toe off (*P* < .001) and ↑ HADD excursion (*P* = .012) in PFP females & PFP RFS compared to controls.
Foch et al., 2020 [46]	30-min treadmill run (5 trials, self-selected speed) Average speed uninjured: 2.9 ± 0.4 m/s Average speed injured: 2.7 ± 0.5 m/s	GMed (surface, anatomical landmarks) GMed (surface, anatomical landmarks)	Filtering: High pass (30 Hz) Smoothing: RMS (50 ms) Normalisation: MVIC Outcome: Muscle on/off > 5 SD above resting mean & mean across loading phase	GMed NS timing and magnitude	No differences in pHADD angle. ↓ HADD excursion in ITBS (*p* = .009)
Higashihara et al., 2019 [48]	40 m overground sprint (3 trials, maximal effort sprint) Average speed: 9.39 ± 0.17 m/s	BF & GMax (bilateral, SENIAM guidelines)	Filtering: High Pass (20 Hz) Smoothing: RMS (10 ms) Normalisation: Sprint max Outcome: Mean across phases of gait cycle	↓ BF activation in injured leg late swing (*p*<.05). GMax: NS.	Pelvic anterior tilt angle ↓ in the previously injured limb during the late-stance phase (p-value = of 0.039). The injured limb exhibited ↑ knee flexion angle during the late-swing phase (p-value= 0.002).
Muniz et al., 2023 []^35^	Treadmill (6 min, 3 speeds) Although 3 different speeds, 3.61 m/s (13 km/h) was selected for Meta-Analysis as this speed was closest representative of the average speed value from rest of papers in review (3.63 m/s)	VMO, VL, GMed (surface, SENIAM)	Filtering: High pass (20 Hz) Smoothing: Rectified & low pass filter (10 Hz) Normalisation: Peak across stance Outcome: Muscle on/off using TKEO*, activation duration & integral across stance	Delayed VMO onset in PFP group (*p*<.05) & shorter duration (*p*<.05). VL: NS. GMed: NS.	↑ HIR at 13 km/h (*p* = .025), ↑ HIR at 11 km/h (*p* = .045) and 13 km/h (*p* = .049), ↓ KADD (*p* = .017 at 9 km/h, *p* = .025 at 11 km/h, *p* = .023 at 13 km/h).
Naderi et al., 2020 [47]	15 m indoor runway (3 trials, 2.68 m/s) Average speed not reported but reported tolerance of ± 5% was allowed	SOL, TA (placement not detailed)	Filtering: High pass (20 Hz) Smoothing: RMS (50 ms) Normalisation: MVIC Outcome: Time of peak, peak and mean across specific gait phases	↑ soleus RMS in MTSS group during the absorption (heel contact to foot flat) (*p* = .01) and propulsion phase (heel lift to toe off) (*p* = .02). TA NS	Participants with MTSS exhibited ↑ BMI (*p* < .001), ↓ VO2max (*p* < .01) and ↓ vigorous physical activity (*p* < .001)
Pingel et al., 2013 [38]	1-hour treadmill run (self-selected speed) Average speed not reported	MG, LG (surface, SENIAM)	Filtering: High pass (3 Hz) Smoothing: No reported Normalisation: None Outcome: Signal frequency (Hz), peak-to-peak amplitude (V) and RMS using Chart analysis software	MG ↑ amplitude in healthy group post-run (*p*<.0.001).	Injured group showed no significant post-run changes.
Smith et al., 2013 [43]	25 m track (6 trials, 4 m/s) Average speed not reported but reported tolerance of ± 10% was allowed. 25 m track (6 trials, 4 m/s)	GMed, GMax (bilateral, SENIAM)	Filtering: High pass (10 Hz) Smoothing: Rectified & moving average (20 ms) Normalisation: None Outcome: Muscle on/off > 15% peak amplitude + visual inspection	GMed: delayed onset (*p*<.001) and shorter duration (*p*<.001) GMax: delayed onset (*p*=.008) shorter duration (*p*=.002) and earlier offset (*p*=.001)	N/A
Souza & Powers, 2009 [37]	15 m lab track (3 trials, 3 m/s) Average speed not reported	GMed, GMax (surface, anatomical landmarks)	Filtering: High pass (35 Hz) Smoothing: Rectified & moving average (75 ms) Normalisation: MVIC Outcome: Mean across stance phase	↑ GMax activation in PFP group (*p*<.05). GMed: NS.	↑ HIR in PFP group (p = < 0.05). ↓ isometric hip abductor strength in PFP (*p* = .2)
Willson et al., 2011 [36]	30 m runway (5 trials, 3.5 m/s) Average speed not reported	GMed, GMax (surface, SENIAM)	Filtering: High pass (30 Hz) Smoothing: Rectified & low pass filter (6 Hz) Normalisation: MVIC Outcome: Muscle on/off > 5 SD resting mean for > 25 ms, duration, peak & mean activity across specific gait phase	Delayed GMed (*p* = .028), shorter duration (*p* = .01) in PFP Gmax NS	Correlation between onset of GMax & GMed with HADD excursion. Correlation between GMax onset & HIR. Correlation between onset of GMax & GMed with HADD excursion. Correlation between GMax onset & HIR. Correlation between onset of GMax & GMed with HADD excursion. Correlation between GMax onset & HIR.
Wyndow et al., 2013 [39]	10 m track (6 trials, self-selected speed) Average speed 4 m/s±100ms)	LG, MG, SOL (bilateral, anatomical landmarks)	Filtering: High pass (20 Hz) Smoothing: Rectified & low pass filter (20 Hz) Normalisation: None Outcome: Muscle on/off > 3 SD above trial minimum for ≥ 100 ms then visually checked and adjusted	↑ MG activation during midstance in AT group (*p*<.05). offset of Sol relative to LG was earlier (*p*<.05).	N/A

*EMG*  Electromyography, *MVIC*  Maximal voluntary isometric contraction, *RMS*  Root mean square, *TKEO*  Teager–Kaiser energy operator, *Hz*  Hertz, *SD*  Standard deviation, *ms*  milliseconds, *m/s*  metres per second, *GMax*  Gluteus maximus, *GMed*  Gluteus medius, *TFL*  Tensor fascia latae, *RF*  Rectus femoris, *VL*  Vastus lateralis, *VMO*  Vastus medialis oblique, *BF*  Biceps femoris, *LG*  Lateral gastrocnemius, *MG*  Medial gastrocnemius, *SOL*  Soleus, *TA*  Tibialis anterior, *PL*  Peroneus longus, *NS*  Non-significant

↑ = increased/higher; ↓ = decreased/lower

*p* = *p*-value

### EMG protocol

#### Electrode placement

All studies used surface electrodes to record EMG activity with various muscles tested including biceps femoris (BF), semimembranosus (SM), gluteus Maximus (GMax), gluteus medius (GMed), vastus medialis obliques (VMO), vastus lateralis (VL), vastus intermedius (VI) rectus Femoris (RF), tensor fascia latae (TFL), tibialis anterior (TA), peroneus longus (PL), soleus (SOL), medial gastrocnemius (MG) and lateral gastrocnemius (LG). There was inconsistency in methods used for electrode placement across studies. Eight studies followed SENIAM guidelines [32–36, 42, 43, 48] whilst six papers reported the exact location of muscle area electrodes were applied to, without mentioning any particular guidelines [37–39, 44–46]. Three papers did not mention any guidelines and did not describe the placement of electrodes on the muscles [41, 42, 47].

#### Signal processing

Eight studies normalised their EMG data against maximal voluntary isometric contractions (MVIC) [32, 35–37, 39, 44, 46, 47], eight studies normalised to the peak value across the running cycle [33, 38, 40–43, 45, 48], and one paper normalised to a dynamic task method [34]. Across studies, key EMG processing parameters such as filter type and frequency, sampling frequency, and data window length were also reported.

### Overview of all injured running populations

**Fig. 2:**
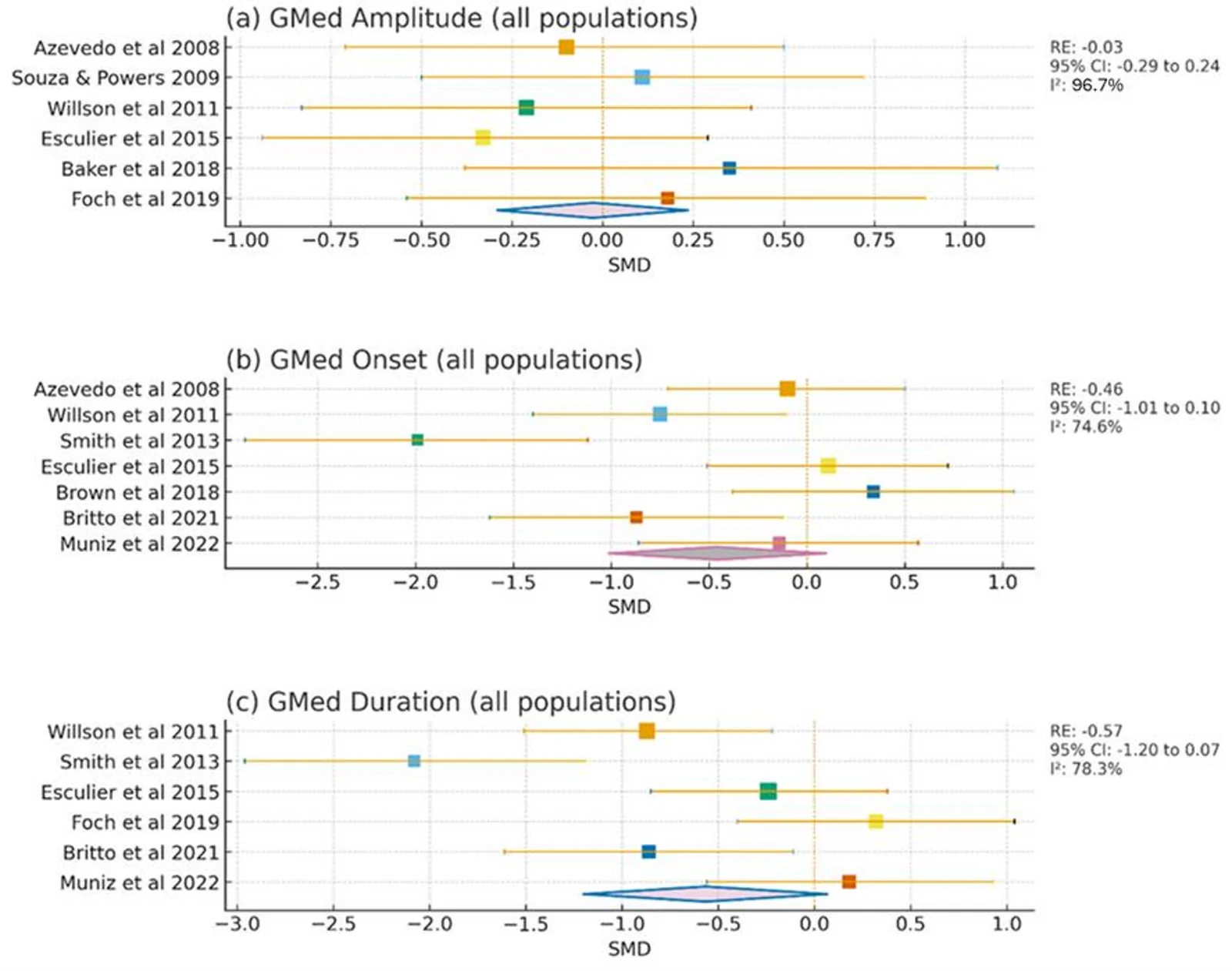
Gluteus medius meta-analyses across all populations. **a** Amplitude, **b** Onset, and (**c**) Duration. Squares represent study effect sizes with 95% confidence intervals; diamonds show pooled random-effects estimates. Negative values indicate lower amplitude, later onset, or shorter duration compared with controls

When examining EMG activity across multiple injury types, only gluteus medius met the predefined criteria for cross-injury meta-analysis (≥ 2 injury types represented) (Fig. 2).

There is strong evidence from three HQ [34, 36, 40] and three MQ studies [37, 44, 46] that GMed amplitude between injured and uninjured runners does not differ (SMD − 0.03, 95%CI -0.29,0.24, *I*^*2*^ 97%).

There is strong evidence from five HQ [33–36, 43] and one MQ studies [46] that the duration of GMed activity between injured and uninjured runners does not differ (non-significant SMD − 0.57, 95% CI -1.24, 0.11, *I²* 78%). There is strong evidence from six HQ [33–36, 40, 43] and one MQ studies [45] that GMed onset between injured and uninjured runners does not differ (non-significant SMD − 0.46, 95% CI -1.01,0.10, *I²* 75%).

### Patellofemoral pain

Six studies (228 participants, 136 females) reported EMG patterns of runners with PFP compared to uninjured runners. Two studies included female-only [36, 37] and two male-only [33, 35] participants. Sufficient data (> 2 studies) were available for meta-analysis of GMed amplitude, onset, and duration, as well as GMax amplitude, VMO, and VL onset and duration of activity.

#### Amplitude

There is strong evidence from two HQ [34, 36] and one MQ studies [37] that GMed amplitude does not differ between runners with PFP and uninjured runners (non-significant SMD − 0.07, 95%CI -0.94, 0.81, *I*^*2*^ 84%).

**Fig. 3:**
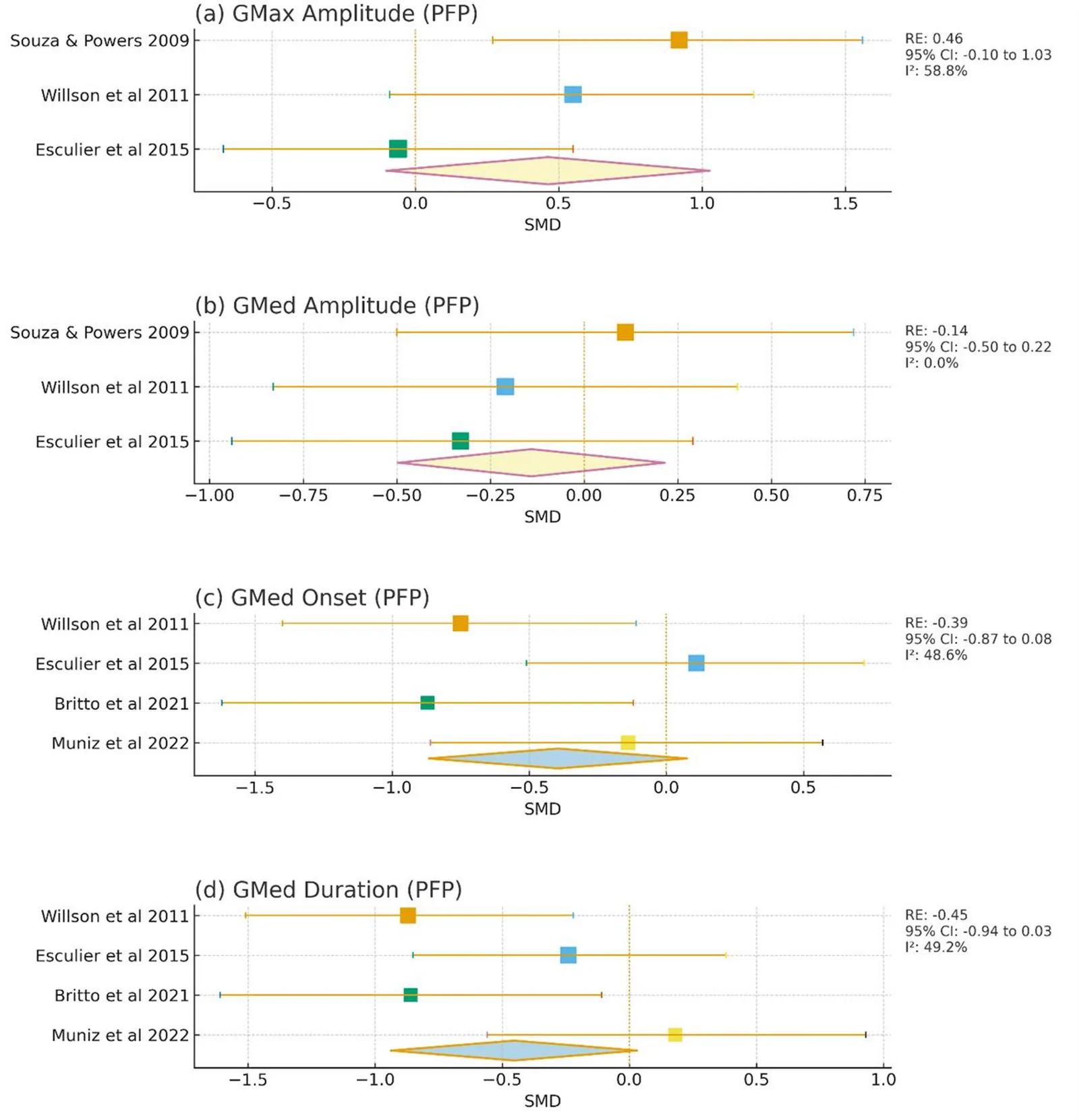
Patellofemoral Pain meta-analyses for gluteus maximus and gluteus medius. **a** Gluteus maximus amplitude, **b** Gluteus medius amplitude, **c** Gluteus medius onset, and (**d**) Gluteus medius duration. Squares show study effect sizes with 95% confidence intervals; diamonds show pooled random-effects estimates. Negative values indicate lower amplitude, later onset, or shorter duration compared with controls

There is conflicting evidence from two HQ [34, 36] and one MQ study [37] regarding GMax amplitude between runners with PFP and uninjured runners (non-significant SMD 0.43, 95%CI 0.70, 1.56, *I*^*2*^ 67%) (Fig. 3).

There is limited evidence from one HQ study [33] that female runners with PFP have reduced VMO activity compared to uninjured runners.

There is limited evidence from one HQ study [35] that male runners with PFP have delayed VMO onset, shorter VMO duration, and no differences in VL onset or duration compared to uninjured runners.

There is strong evidence from two HQ studies [33, 35] that there are no differences in VL or GMed integrals between runners with PFP and uninjured runners.

There is conflicting evidence for VMO integral, with one HQ study [33] reporting reduced VMO integral in runners with PFP and another [35] reporting no differences compared to uninjured runners.

#### Temporal activity

There is strong evidence from four HQ studies [33–36] that GMed onset does not differ between runners with PFP and uninjured runners (non-significant SMD − 0.40, 95%CI -0.07, 0.07, *I*^*2*^ 94%) (Fig. 3).

There is strong evidence from four HQ studies [33–36] that GMed duration does not differ between runners with PFP and uninjured runners (non-significant SMD − 0.45, 95%CI -0.93, 0.03, *I*^*2*^ 94%) (Fig. 3).

**Fig. 4:**
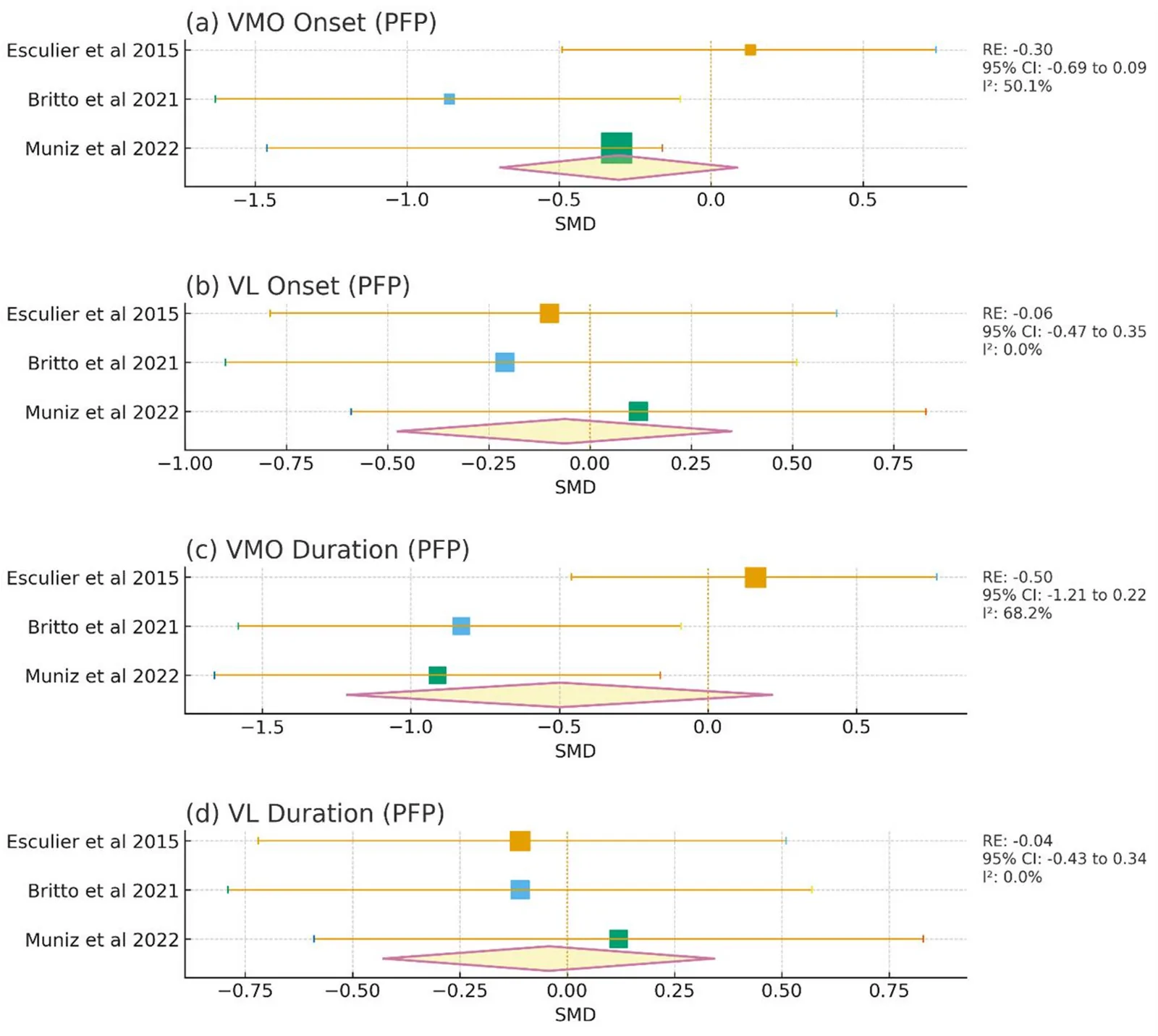
Patellofemoral Pain meta-analyses for vastus medialis oblique and vastus lateralis. **a** Vastus medialis oblique onset, **b** vastus lateralis onset, **c** vastus medialis oblique duration, and (**d**) vastus lateralis duration. Negative values indicate later onset or shorter duration in Patellofemoral Pain compared with controls

There is strong evidence from three HQ studies [33–35] that VMO onset (non-significant SMD − 0.52, 95%CI -2.0, 0.97, *I* 94%), duration (non-significant SMD − 0.50, 95%CI -2.00, 1.00, *I*^*2*^ 94%), and VL onset (non-significant SMD − 0.10, 95%CI -0.54, 0.34, *I*^*2*^ 99%), and duration (non-significant SMD − 0.04, 95%CI -0.36, 0.28, *I*^*2*^ 99%) do not differ between runners with PFP and uninjured runners (Fig. 4).

There is moderate evidence from one HQ study [33] of reduced VMO integral in male runners with PFP compared to uninjured runners.

There is limited evidence from one MQ study [32] of increased Hamstring-Quadriceps co-contraction during the first 60% of the stance phase in runners with PFP compared to uninjured runners.

There is moderate evidence from one HQ study [34] that SOL activity is longer during stance in runners with PFP compared to uninjured runners.

There is moderate evidence from one HQ study [33] of earlier offset times for VMO and GMed, but no differences in VL offset, in runners with PFP compared to uninjured runners.

### Achilles tendinopathy

Six case-control studies (209 participants, 38 females, 171 males) compared muscle activation patterns between runners with AT and uninjured runners. Two studies investigated male-only cohorts [39, 43], three included mixed-sex cohorts [38, 40, 42] and one did not report participant sex or sex ratio [41].

#### Amplitude

There is moderate evidence from one HQ study [40] of reduced TA amplitude prior to heel strike and reduced RF and GMed Integral EMG activity during stance in runners with AT compared to uninjured runners.

There is moderate evidence from one HQ [42] study of reduced PL and MG activity in runners with AT compared to uninjured runners.

There is limited evidence from one MQ study [38] of increased MG activity at the start and immediately following a 1-hour treadmill run in runners with AT compared to uninjured runners.

There is conflicting evidence for LG activity, with one MQ study [38] reporting significantly higher activation in runners with AT and two HQ studies [40, 42] reporting no differences.

#### Temporal activity

There is moderate evidence from one HQ study [43] of delayed GMed onset, earlier offset and shorter duration of activity in runners with AT compared to uninjured runners.

There is limited evidence from one MQ study [39] of earlier SOL offset relative to LG in runners with AT compared to uninjured runners.

### Iliotibial band syndrome

Three studies (92 participants, 59 females) compared EMG patterns of runners with ITBS to uninjured controls. Two studies investigated female-only cohorts [45, 46], while one used a mixed sex cohort [44].

#### Amplitude

There is limited evidence from one MQ study [44] of higher TFL amplitude in runners with ITBS compared to uninjured runners at the start of a treadmill run, but no difference after 30 min of running.

There is moderate evidence from two MQ studies [44, 46] no significant differences in GMed amplitude in runners with ITBS compared to uninjured runners.

#### Temporal activity

There is moderate evidence from three MQ studies [44–46] of no difference in GMed onset in runners with ITBS compared to uninjured runners.

There is limited evidence from one MQ study [44] of no difference in GMax onset following a fatiguing run in runners with ITBS compared to uninjured runners.

There is limited evidence from one MQ study [45] of no difference in TFL onset pre or post a fatiguing run in runners with ITBS compared to uninjured runners.

### Medial tibial stress syndrome

One HQ prospective cohort study [47] compared EMG patterns in runners that developed MTSS compared to uninjured runners and there is moderate evidence of greater SOL amplitude in runners who subsequently developed MTSS compared to those who remained uninjured.

### Hamstring strain injury

One MQ study [48] compared between-limb EMG patterns in runners with a history of HSI. There is limited evidence of reduced BF amplitude during late swing in the injured limb compared to the uninjured limb, but no difference in GMax amplitude [48].

## Discussion

### Main findings

This systematic review aimed to investigate whether EMG patterns differed amongst injured and uninjured runners. Across the various injuries included, only GMed activity had sufficient data available for pooling across studies, with strong evidence of no difference in amplitude, duration, or timing of muscle onset. PFP was the only individual injury with sufficient data for pooling, with strong evidence indicating no difference in the amplitude of GMax or GMed activity, and no difference in the onset or duration of GMed, VMO or VL activity. These findings suggest that neuromuscular deficits are not consistently observed across or within specific running injury types.

The findings of the present review contrast with those of an earlier systematic review by Semciw et al., [24], which reported associations between GMed muscle activity and running-related injuries. Specifically, Semciw et al., [24] identified a shorter duration of GMed activity in individuals with PFP compared to controls. Their meta-analysis only included data from two studies [34, 36], whereas the current review expands on this by incorporating two additional studies [33, 35], providing a more robust summary of the existing evidence. The findings of our meta-analyses on GMed parameters challenge previous conclusions that GMed function is impaired in individuals with PFP and may indicate that interventions specifically targeting GMed recruitment are not appropriate. While Semciw et al., [24] also noted limited evidence of altered muscle activity in AT populations, heterogeneous and/or insufficient data prevented data pooling for AT and other running-related injuries, which aligns with the findings of the current review. This limits the collective ability to infer whether alterations in muscle activity are present within and across running injuries.

Only one prospective study was identified [47], with the remaining studies being predominantly retrospective case-control investigations. This limits our ability to establish causal inferences about the role of EMG in injury development. It has been proposed that the pain following injury may lead to individual-specific adaptations in muscle activity [23]. Deficits identified in retrospective studies may therefore represent consequences of injury rather than predisposing factors. Understanding such adaptations may still have important implications for injury management and rehabilitation. In the short term, alterations in muscle activity may serve to protect the injured region by offloading tissue and limiting pain provocation. If these patterns persist, they may result in chronic underloading of affected structures and contribute to delayed recovery or recurrent injury [23, 49]. However the lack of consistent evidence for a specific direction of EMG change across running injuries makes the role of neuromuscular adaptations uncertain, limiting our ability to direct interventions towards muscle activity.

### Limitations of the existing literature

Significant methodological heterogeneity was present in the existing literature, which results in limited data synthesis and thus inconsistent results. High between-study heterogeneity was evident, with *I²* values frequently exceeding 75% in pooled analyses. Methodological inconsistencies in EMG protocols limit comparability, with substantial variation in normalisation tasks (e.g., MVIC, task-peak, dynamic), onset detection methods (e.g., SD thresholds, Teager–Kaiser), reporting metrics (milliseconds vs. percentage of gait cycle), running speed (fixed versus self-selected), and the use of fatigued versus non-fatigued testing conditions. These differing methodologies may directly influence the magnitude, timing, and clinical interpretation of reported EMG parameters. Differences in task specificity, contraction intensity, and biomechanical constraints imposed by testing protocols can alter the neural and mechanical demands placed on a muscle, thereby influencing conclusions regarding the “overactivity” or “underactivity” of a muscle. For instance, while Willson et al., [36] and Baker et al., [44] used similar MVC protocols for GMax, they reported markedly different EMG amplitude (31% and 81.4%, respectively), which may have stemmed from differences in running task conditions (30-meter indoor track compared to treadmill running). Esculier et al., [34] normalised GMed activity to a prone hip extension task with a 2 kg ankle weight, reporting peak activation of 165% MVIC during running. In contrast, Souza and Powers [37] used a side-lying MVC task with a fixed strap, reporting only 28% MVIC in the same muscle. Such discrepancies highlight how normalisation methods may influence EMG parameters, leading to different interpretations of muscle function during running, even when examining the same population and muscle group.

Protocols for calculating onset time were also inconsistent. Two studies employed a Teager–Kaiser Energy Operator [33, 35] while others used threshold-based definitions, such as exceeding five standard deviations above the resting mean for 25ms [34, 36], exceeding 15% of peak amplitude for more than 10% of gait cycle [38], or exceeding 10% of the difference between maximum activity and resting mean [45]. Onset was also reported either as a percentage of the gait cycle or as an absolute time in milliseconds. This is problematic as absolute time is influenced by running speed and stride and swing duration, whereas percentage values may obscure the actual time delay in activation. Prior studies have shown different onset detection methods can produce substantial variation in the estimated onset time [22, 50, 51]. Consequently, inconsistent onset definitions risk misclassifying normal temporal variability as pathological delay or compensatory strategy. This lack of a universally accepted normalisation or onset detection method prevents reliable cross-study comparisons and likely contributes to the wide variability in reported outcomes.

Electrode placement protocols also varied between studies. While most studies followed SENIAM guidelines [32–36, 42, 43, 48], others provided insufficient detail to determine their electrode placement [37–40, 44–46], and three studies did not report electrode placement at all [41, 42, 47]. Inconsistent or unverified placement can introduce measurement error and reduce data repeatability, particularly due to cross-talk from neighbouring muscles or positioning too close to the innervation zone [52]. Specifically, electrodes placed across the innervation zone may capture phase cancellation, reduced amplitude, or unstable waveforms, leading to underestimation of muscle activity and increased variability across trials and participants [52]. Crosstalk from adjacent muscles can also contaminate the signal and obscure muscle-specific activation patterns [53]. Thus, it is important to improve methodological consistency in electrode placement to minimise the likelihood that observed differences in EMG parameters are related to differences in placement.

The scope of muscle assessment was also frequently narrow. Only three of the 17 studies examined five or more muscles, with most focusing on three or fewer. Many restricted analysis to either proximal (e.g., gluteal, hamstring) or distal (e.g., gastrocnemius, tibialis anterior) muscles, with few exploring both regions in a single protocol. This limits the ability to characterise whole-limb neuromuscular coordination during running, which may provide important insights for understanding injury mechanisms.

Participant characteristics were inconsistently reported. Variables such as running experience, weekly mileage, and prior injury history were often omitted from studies, and in some cases participants were included despite being symptom-free at the time of testing [46, 48]. Completing high weekly running mileages has been shown to reduce RF and hamstring muscle activity compared to lower mileage runners [54], while another study reported higher gastrocnemius activity during stance phase in low mileage runners [55]. Finally, all the included studies used surface EMG, and while this is the most common method of application, it is subject to cross talk and the overall findings may not therefore represent the true underlying muscle function. These findings highlight that methodological variability may influence EMG patterns and highlights the need for consistent reporting and standardisation of participant characteristics within running biomechanics studies.

### Future research directions

Future research should prioritise integration of EMG from multiple muscles with whole-body kinematic and kinetic measurements. Although the present review found no consistent differences in isolated EMG parameters between injured and uninjured runners, there is evidence that several running injuries are associated with repeatable kinematic and kinetic patterns [9–14]. The absence of consistent, injury-specific EMG changes in the current review may suggest that it is difficult to interpret muscle activation patterns in isolation without concurrent kinetic and kinematic data. Future studies should therefore integrate EMG from multiple lower limb muscles with full lower-limb and trunk kinematics and kinetics. This would provide insight on whether injury-related differences reflect altered whole-body neuromuscular control strategies rather than isolated muscle dysfunction or adaptation.

Future prospective longitudinal studies are also required. This will provide insight into whether potential observed differences in muscle activation precede injury onset or represent adaptations secondary to pain and tissue pathology. Current literature is dominated by retrospective case–control designs, with only one prospective study identified to date [47]. This limits our ability to determine whether altered neuromuscular patterns precede injury or represent adaptations following symptom onset [23, 49]. Addressing this gap will allow researchers to determine whether rehabilitation should target predisposing coordination patterns or focus on secondary adaptations.

Greater standardisation in EMG methodology is required to advance research into injury mechanics. As highlighted above, there is substantial inconsistency in testing protocols and EMG parameters reported in previous studies investigating running. To address this limitation, researchers should establish university-accepted guidelines for the collection and analysis of EMG data in running, including running speeds, electrode placement, normalisation methods and derivation of EMG outcome parameters. This would improve reporting and facilitate evidence synthesis across multiple studies.

### Strengths and limitations

To the best of our knowledge, this is the first systematic review to analyse all relevant literature exploring EMG patterns in injured runners. A strength of this review is its broad clinical scope, synthesising EMG findings across multiple common running injuries, including PFP, AT, ITBS, MTSS, and HSI. This approach allows for comparisons both within specific conditions and across different injury types, providing insights into whether neuromuscular adaptations are shared or condition-specific. The comprehensiveness of the search strategy further strengthens the review, with six major databases searched alongside citation tracking. Screening was conducted systematically, with duplicates removed and study eligibility assessed by two independent reviewers ensuring methodological rigour and minimising selection bias.

This review has several limitations. First, the search terms used were limited and did not include Medical Subject Headings (MeSH), which may have reduced the precision and recall of studies using alternative terminology or spelling variations. To mitigate this, hand searching was performed through the citation lists and reference sections of included studies. Second, data extraction was conducted by a single reviewer and verified through a secondary check, rather than by two independent extractors, which may introduce a minor risk of bias or human error in data interpretation.

## Conclusion

This systematic review revealed no significant differences in EMG amplitude, onset, or duration for key lower-limb muscles in injured populations (PFP, AT, ITBS, MTSS and HSI), including GMed across all and PFP-specific cohorts, and GMax, VMO, and VL within PFP populations. For other muscles and injury groups evidence could not be pooled and, although isolated findings were reported, the available data were insufficient to support consistent or robust differences in muscle activation between injured runners and healthy controls. These results may be influenced by methodological heterogeneity such as differences in electrode placement, EMG normalisation, task protocols, participants recruited and individual kinematic patterns. Future research should aim to use standardised EMG protocols, consider a wider range of lower limb muscles, and appropriately report details of the running populations included.

## Supplementary Information

Supplementary Material 1.

Supplementary Material 2.

## Data Availability

The datasets generated and analysed during the current study are available from the corresponding author on reasonable request.
