# Supplementary material for: Electromyography patterns during running in injured versus healthy runners: a systematic review
Source: BMC Musculoskelet Disord. 2026 Jul 9;27:737. doi: 10.1186/s12891-026-10064-z (PMC13520374; doi:10.1186/s12891-026-10064-z)
Supplement: Supplementary file 1 [file 12891_2026_10064_MOESM1_ESM.docx]

# Supplementary File 1. Full Electronic Search Strategies

This supplementary material provides the complete electronic search strategies used for each database, including search fields, Boolean logic, truncation, and applied filters. Searches were conducted from database inception to February 2025.

## Supplementary Table S1. Database-specific search strategies

| Database | Platform | Search fields | Search strategy | Filters applied |
| --- | --- | --- | --- | --- |
| MEDLINE | PubMed | Title and Abstract | Electromyography OR electromyo* OR EMG combined with run* using Boolean AND. Controlled vocabulary for electromyography was included where available and combined with free-text terms. | Humans; Adults (≥18 years); English language; no publication date or running speed restrictions |
| CINAHL | EBSCOhost | Title and Abstract; CINAHL Headings | Electromyography OR electromyo* OR EMG combined with run* using Boolean AND. CINAHL subject headings for electromyography were included and combined with free-text keyword searches. | Adults; English language; no publication date or running speed restrictions |
| Scopus | Elsevier | Title, Abstract, and Keywords | Electromyography OR electromyo* OR EMG combined with run* using Boolean AND. Searches were limited to title, abstract, and keyword fields. | English language; no publication date or running speed restrictions |
| Web of Science | Clarivate Analytics | Topic field (Title, Abstract, Author Keywords, Keywords Plus) | Electromyography OR electromyo* OR EMG combined with run* using Boolean AND. Searches were conducted using the Topic field. | English language; no publication date or running speed restrictions |
| ProQuest | ProQuest Central | Title and Abstract | Electromyography OR electromyo* OR EMG combined with run* using Boolean AND. Searches were limited to title and abstract fields. | English language; no publication date or running speed restrictions |
| Google Scholar | Google | Full text | Reference list screening and citation tracking were also performed for all included studies. | English language; results screened by relevance |
