# Supplementary material for: Electromyography patterns during running in injured versus healthy runners: a systematic review
Source: BMC Musculoskelet Disord. 2026 Jul 9;27:737. doi: 10.1186/s12891-026-10064-z (PMC13520374; doi:10.1186/s12891-026-10064-z)
Supplement: Supplementary file 2 [file 12891_2026_10064_MOESM2_ESM.docx]

|  | **Reporting** | | | | | | | **External Validity** | | **Internal Validity** | | | | **Selection bias** | |  | | | | |
| --- | --- | --- | --- | --- | --- | --- | --- | --- | --- | --- | --- | --- | --- | --- | --- | --- | --- | --- | --- | --- |
|  | 1 | 2 | 3 | 5 | 6 | 7 | 10 | 11 | 12 | 15 | 16 | 18 | 20 | 21 | 25 | TOTAL | RATING |  |  |  |
| **Azevedo et al., 2009** | 1 | 1 | 1 | 1 | 1 | 1 | 1 | 0 | U | U | 1 | 1 | 1 | U | 1 | 11 | HQ |  | HQ | 8 |
| **Baker et al., 2018** | 1 | 1 | 1 | 1 | 1 | 1 | 1 | U | U | U | 1 | 1 | 1 | U | 0 | 10 | MQ |  |  |  |
| **Baur et al., 2004** | 1 | 1 | 0 | 0 | 0 | 0 | 0 | 0 | U | U | 1 | 1 | U | U | 0 | 4 | LQ |  | MQ | 8 |
| **Baur et al., 2011** | 1 | 1 | 1 | 2 | 0 | 1 | 1 | 1 | U | U | 0 | 1 | 1 | 1 | 1 | 12 | HQ |  | LQ | 1 |
| **Besier et al., 2009** | 1 | 1 | 0 | 1 | 1 | 1 | 1 | 0 | U | U | 1 | 1 | 0 | U | 0 | 8 | MQ |  |  |  |
| **Brown et al., 2019** | 1 | 1 | 0 | 2 | 1 | 1 | 1 | U | U | U | 1 | 1 | 1 | U | 0 | 10 | MQ |  |  |  |
| **De Almeria Britto et al., 2021** | 1 | 1 | 1 | 2 | 0 | 1 | 1 | 1 | U | U | 1 | 1 | 1 | U | 1 | 12 | HQ |  |  |  |
| **Esculier et al., 2015** | 1 | 1 | 1 | 2 | 1 | 1 | 1 | 0 | U | U | 1 | 1 | 1 | U | 1 | 12 | HQ |  |  |  |
| **Foch et al., 2020** | 1 | 1 | 0 | 2 | 1 | 1 | 1 | U | U | U | 1 | 1 | 1 | U | 0 | 10 | MQ |  |  |  |
| **Higashihara et al., 2019** | 1 | 1 | 1 | 1 | 1 | 0 | 1 | U | U | U | 1 | 1 | 0 | 1 | 0 | 9 | MQ |  |  |  |
| **Muniz et al., 2023** | 1 | 1 | 1 | 2 | 1 | 1 | 1 | 0 | U | U | 1 | 1 | 1 | 1 | 1 | 13 | HQ |  |  |  |
| **Naderi et al., 2020** | 1 | 1 | 1 | 2 | 1 | 1 | 1 | 1 | 1 | U | 0 | 1 | 1 | 1 | 1 | 14 | HQ |  |  |  |
| **Pingel et al., 2013** | 1 | 1 | 0 | 0 | 0 | 1 | 1 | 0 | U | U | 1 | 1 | 1 | U | 1 | 8 | MQ |  |  |  |
| **Smith et al., 2013** | 1 | 1 | 1 | 2 | 1 | 1 | 1 | 1 | U | U | 1 | 1 | 1 | 1 | 1 | 14 | HQ |  | |  |
| **Souza & Powers, 2009** | 1 | 1 | 0 | 1 | 0 | 1 | 1 | 0 | U | U | 1 | 1 | 1 | U | 1 | 9 | MQ |  |  |  |
| **Willson et al., 2011** | 1 | 1 | 1 | 2 | 1 | 1 | 1 | U | U | U | 1 | 1 | 1 | 1 | 0 | 12 | HQ |  |  |  |
| **Wyndow et al., 2013** | 1 | 1 | 1 | 2 | 0 | 1 | 0 | 0 | U | U | 1 | 1 | 1 | 1 | 0 | 10 | MQ |  |  |  |
